# Transcription and translation of *APOL1* variants

**DOI:** 10.1042/BSR20170647

**Published:** 2017-10-11

**Authors:** Samina Ejaz

**Affiliations:** Department of Biochemistry and Biotechnology, The Islamia University of Bahawalpur, Bahawalpur, Pakistan

**Keywords:** Apolipoprotein, genetic variants, kidney diseases, transcription, translation

## Abstract

It is highly important to document the molecular alterations existing in normal cells prior to the onset of any disease. Knowledge of genetic mutations and associated molecular mechanisms will be helpful for better diagnosis and management of disease. The major focus of this commentary on providing understanding about the *apolipoprotein 1* (*APOL1*) gene, the protein encoded by this gene (apoL1) and the mechanistic details regarding the role of apoL1 in the lysis of *Trypanosoma brucei*. Information about *APOL1* genetic variants, *APOL1G1* and *APOL1G2*, is provided along with the association of these variants with hypertension-attributed end-stage renal disease (ESRD) and focal segmental glomerulosclerosis (FSGS). Moreover, this commentary presents a brief overview of how the authors of a recent *Bioscience Reports* article [Haque et al (2017) **37**, BSR20160531, doi: 10.1042/BSR20160531] have evaluated the functional impact of G1 and G2 alleles on the transcription and translation of *APOL1* mRNA.

Long before the onset of any disease, the microenvironment in a normal cell starts changing to promote transformation of the normal cell into a diseased cell. Such changes in the cellular environment serve as indicators of the prevailing physiological condition. Measureable cellular, biochemical or molecular alterations that reflect the physiological state of the biological medium are known as biomarkers [16].

A biomarker is a molecular entity like a gene, RNA or protein present in the cell. It varies in response to a stimulus and alters the physiological state of cell. Biomarkers are not only helpful in diagnosing disease as early as possible, but also facilitate monitoring of the disease prognosis and outcome of treatment [16]. Usually disease is not the outcome of abnormality in a single gene, RNA or protein. Most diseases are multifactorial and arise due to combined effects of multiple dysregulated molecular processes [22]. Molecular processes are multistep and involve contributions of many functional molecules, such as RNA and proteins. Each functional molecule is encoded by a specific gene. A gene is said to be expressed when a functional molecule is synthesized by decoding information contained in the gene. The process of gene expression for protein-coding genes consists of four stages: transcription, post-transcriptional processing, translation and post-translational modifications (Figure 1). Transcription takes place inside the nucleus and synthesized pre-mRNA undergoes capping (addition of a guanosine cap at the 5′ end), splicing (removal of non-coding regions and joining of coding regions) and polyadenylation (addition of polyA tail at the 3′ end) before it is transported to cytosol for translation. In the cytosol, translational machinery, consisting of ribosomes and accessory proteins, attaches to mRNA to decode the codons of mRNA into the amino acid sequence of proteins. Nascent proteins undergo post-translational modifications such as phosphorylation, acetylation and proteolytic cleavage to be converted into a functionally active protein and targeted to its proper destination [13,14].

**Figure 1:**
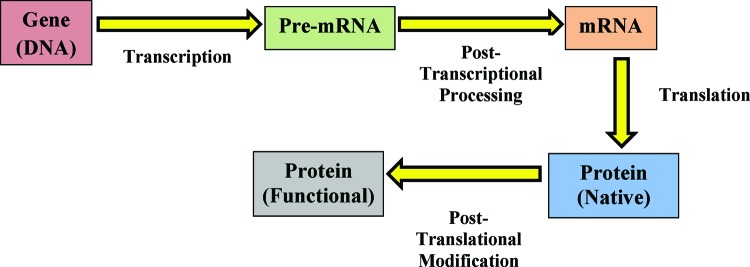
Steps involved in eukaryotic gene expression process

Each level of gene expression is mediated by specific macromolecular machinery and regulated by distinct *cis*- and *trans*-acting factors. *Cis*-acting factors include sequence elements present in DNA and RNA, while *trans*-acting factors are proteins and regulatory RNAs binding to these sequence elements. The difference between *cis*- and *trans*-acting factors can be best explained by the example of RNA polymerase, which acts as a *trans*-acting factor, and its binding site in DNA, the *cis*-acting factor. Various *cis*- and *trans*-acting factors are known and gene expression processes are tightly regulated. Some factors are stimulatory while others are inhibitory. Similarly, some act synergistically whereas others antagonize the effects of each other. The net effect is determined by the balance between stimulatory and inhibitory factors. The distribution of *cis*- and *trans*-acting factors differs in different tissues and this difference in the nature and concentration of *trans*-acting factors helps to maintain tissue-specific gene expression [2,4,17]. In addition to factors operating at a genetic level, epigenetic mechanisms like DNA methylation and acetylation regulate chromatin structure and the pattern of gene expression [12].

The pattern of gene expression is altered due to changes in the sequence of genes (*cis*-acting elements) and modulated activity of regulatory molecules (*trans*-acting elements). Similarly, any alteration in chromatin structure achieved through epigenetic mechanisms can cause stable alteration in the pattern of gene expression [2,12,13]. Any abnormality or variation in the gene is technically termed as a mutation and it may the modify sequence, function or yield of the encoded molecule. A wide range of mutations including insertion, deletion, substitution, frame shift, missensense and non-sense mutations are known [5,14]. Mutations are induced by a variety of mutagens, such as exposure to radiant energy, chemicals and biological factors like viruses [14,23].

Mutations in DNA if present in the regulatory regions may affect the level of gene expression by compromising the rate of transcription or translation. However, mutations in the protein-coding regions may lead to the synthesis of mutated proteins with altered amino acid sequences. Sometimes the alteration in a single amino acid causes a drastic change in the structure of protein and ultimately, function of the protein is either lost or altered. Proteins are the main players inside cells, regulating different physiological process and must be present at specific cellular concentrations. Any abnormality in the function of the protein or level of protein disrupts the associated physiological process and results in a physiological disorder [1,3,4,9,25].

It is extremely beneficial to screen disease cells and document abnormalities occurring at the molecular level, as this may serve as a signature of disease. Molecular level abnormalities are helpful in identifying the risk of developing a disease and determining genetic predisposition of a disease. In addition to this, sometimes changes at the molecular level are used as tools to study the course of disease and monitor treatment [7,24]. Documenting disease-associated abnormalities enables investigators to understand what is happening in the disease, and knowing how and why it is happening are rather priority questions addressed by scientific community.

Such an attempt was made by Haque et al. in a recent *Bioscience Reports* article entitled ‘Effect of *APOL1* disease risk variants on *APOL1* gene product’ [10]. They evaluated the impact of mutations in *APOL1* genetic variants on transcription and translation of *APOL1*. Apolipoprotein A1 (apoL1) is a protein encoded by the *APOL1* gene located on chromosome 22 at position q12.1–q13.1 22. *APOL1* is 14-kb gene which consists of seven exons and present in a cluster (spanning 127 kb) with three other members of apolipoprotein family, *APOL2, APOL3* and *APOL4* [6]. *APOL1* encodes a 43.9 kDa protein containing 398 amino acids and five functional domains [20]. ApoL1 constitutes the apoprotein part of high-density lipoprotein (HDL) and is the human-specific serum apolipoprotein bound to HDL particles [21]. It is synthesized in many tissues of the human body including liver, kidney, pancreas and brain. Similarly, it is present in various parts of the human body including liver, lung, proximal tubule, placenta, heart, podocytes and arterial cells. The secreted form of this protein is also present in the blood and confers a trypanosome lytic capability to human serum [15,18,21]. ApoL1 protein forms a protein complex with HDL3 particles which contain two other proteins, i.e. apolipoprotein A1 and the hemoglobin-binding, haptoglobin-related protein (HPR). This multiprotein complex known as trypanosome lytic factor-1 (TLF1) is supposed to protect against *Trypanosoma brucei* infection [11,27]. Moreover, it has been demonstrated that the membrane pore-forming domain of apoL1 functionally resembles the bacterial toxins colicins and induces the formation of anion channels in the lipid bilayer of biological membranes. Upon entry of *T. brucei*, apoL1 is targeted to the lysosomal membrane and causes its depolarization. Depolarization of the lysosomal membrane promotes continuous influx of chloride ions, disturbs osmotic concentration and keeps enhancing osmotic swelling of the lysosome until the trypanosome is lysed [21,26].

In east Africa, human sleeping sickness is caused by the parasite *T. brucei rhodesiens*, which is resistant to lysis by human serum. *T. brucei rhodesiens* develops this resistance due to the expression of a modified or truncated isoform of a surface glycoprotein known as serum-resistance-associated protein (SRA) present in the lysosomes. Through endocytic pathways, apoL1 is taken up and reaches the lysosomes. It has been shown that the N-terminal α-helix of the variant SRA interacts with the C-terminal α-helix of apoL1 resulting in the complete loss of apoL1's trypanosome lysis ability [26].

A 2010 study demonstrated the existence of two independent sequence variants of the *APOL1* gene in African-Americans and association of these variants with hypertension-attributed end-stage renal disease (ESRD) and focal segmental glomerulosclerosis (FSGS) [8]. These genetic variants of *APOL1* were termed as *APOL1G1* and *APOL1G2* and were only noticed in African-Americans. None of these variants was present in European populations. The *APOL1G1* allele contains two non-synonymous coding variations localized in the last exon of *APOL1* which leads to the substitution of two amino acids: Ser^342^ to glycine and Ile^384^ to methionine. However, the *APOL1G2* allele harbours a six-base-pair deletion that causes deletion of two amino acids (Asn^388^ and Tyr^389^) in the encoded protein. Moreover, both alleles, *APOL1G1* and *APOL1G2*, are mutually exclusive, follow completely recessive pattern of inheritance and rarely undergo genetic recombination [8].

*In vitro* assays were performed to reveal the functional impact of these genetic variations on encoded proteins. Results indicated that the apoL1 proteins encoded by only kidney disease-associated variants possess the ability to lyse *T. brucei rhodesiens*. Investigators proposed that this may be due to the evolution of a critical survival factor that contributes to the higher prevalence rate of renal disease in African-Americans [8].

The study of Haque et al. [10] continued work exploring the effects of *APOL1G1* and *APOL2G2* on downstream molecular processes like transcription and translation. It is known that genetic mutations can cause variation in the pattern of transcription and translation, the stability of the encoded protein and the way in which the protein folds. Therefore, they hypothesized that genetic mutations noticed in the *APOL1* variants G1 (substitution mutation) and G2 (deletion mutation) might affect the transcription and translation of *APOL1* mRNA. To study the effect on expression level, they transfected HEK239T cells with *APOL1* empty vector, *APOL1G0* (wild-type), *APOL1G1* and *APOL2G2* plasmids. Protein expression was determined by Western blotting and significantly lowered expression was observed in case of *APOL1G1* and *APOL2G2* variants as compared with *APOLIG0*. Apol1 expression was also studied in proliferating and differentiated human podocytes through Western blotting and FACS analysis. Both types of cell exhibited lowered expression of *APOL1G1* and *APOL1G2* variants as compared with *APOL1G0*. The degree of expression varied in the following order: *APOL1G0* > *APOL1G1* > *APOL2G2*. In accordance with apoL1 protein expression, *APOL1* mRNA levels were also decreased in proliferating and differentiated human podocytes. It was further observed that within a period of 0.5–3 h, a considerable fraction of *APOL1G1* mRNA (10–15%) and *APOL1G2* mRNA (15–20%) is degraded. In the light of their observations, Haque et al. [10] have concluded that lowered expression level of apoL1 protein encoded by *APOL1G1* and *APOL2G2* genetic variants is the outcome of a decreased rate of transcription and enhanced degradation rate of the encoded transcripts.

The preliminary study by Haque et al. [10] has evaluated functional impact of kidney disease-associated *APOL1* genetic variants on the expression of the *APOL1* gene. Further study is required to understand the underlying molecular mechanisms that contribute to altered expression of the *APOL1* gene. This approach enhances understanding of key target molecules and helps to design better therapeutic drugs and treatment strategies for managing diseases [19].

## Abbreviations

APOL1: apolipoprotein 1
HDL: high-density lipoprotein
HEK: human embryonic kidney
